# First clinical experience in 14 patients treated with ADVOS: a study on feasibility, safety and efficacy of a new type of albumin dialysis

**DOI:** 10.1186/s12876-017-0569-x

**Published:** 2017-02-16

**Authors:** Wolfgang Huber, Benedikt Henschel, Roland Schmid, Ahmed Al-Chalabi

**Affiliations:** 1 II. Medizinische Klinik und Poliklinik, Klinikum rechts der Isar, Technische Universität München, Ismaninger Straße 22, D-81675 Munich, Germany; 2 Klinik für Anaesthesiologie der Universität München, Campus Großhadern, Marchioninistraße, 15 81377 Munich, Germany; 3 Jamaica Hospital Medical Center, 8900 Van Wyck Expy, Jamaica, NY 11418 USA

**Keywords:** Chronic liver failure, Acute-on-chronic-liver failure, Acute liver failure, Extracorporeal liver support, Liver transplantation, Single pass albumin dialysis, Molecular Adsorbent Recirculating System, Fractionated plasma separation and adsorption, CLIF-SOFA, MELD

## Abstract

**Background:**

Liver failure (LF) is associated with prolonged hospital stay, increased cost and substantial mortality. Due to the limited number of donor organs, extracorporeal liver support is suggested as an appealing concept to “bridge to transplant” or to avoid transplant in case of recovery. ADVanced Organ Support (ADVOS) is a new type of albumin dialysis, that provides rapid regeneration of toxin-binding albumin by two purification circuits altering the binding capacities of albumin by biochemical (changing of pH) and physical (changing of temperature) modulation of the dialysate.

It was the aim of this study to evaluate feasibility, efficacy and safety of ADVOS in the first 14 patients ever treated with this procedure.

**Methods:**

Patients included suffered from acute on chronic LF (*n* = 9) or “secondary” LF (*n* = 5) which resulted from non-hepatic diseases such as sepsis. The primary endpoint was the change of serum bilirubin, creatinine and serum BUN levels before and after the first treatment with ADVOS. The Wilcoxon Signed Rank test for paired samples was used to analyze the data.

**Results:**

A total of 239 treatments (1 up to 101 per patient) were performed in 14 patients (6 female, 8 male). Mean age 54 ± 13; MELD-score 34 ± 7; CLIF-SOFA 15 ± 3. Serum bilirubin levels were significantly decreased by 32% during the first session (26.0 ± 15.4 vs. 17.7 ± 10.5 mg/dl; *p* = 0.001). Similarly, serum creatinine (2.2 ± 0.8 vs. 1.6 ± 0.7 mg/dl; *p* = 0.005) and serum BUN (49.4 ± 23.3 vs. 31.1 ± 19.7 mg/dl; *p* = 0.003), were significantly lowered by 27% and 37%, respectively. None of the treatment sessions had to be interrupted due to side effects related to the procedure.

**Conclusion:**

ADVOS efficiently eliminates water- and protein-bound toxins in humans with LF. ADVOS is feasible in patients with advanced LF which is emphasized by a total number of more than 100 treatment sessions in one single patient.

## Background

Based on its course, liver failure (LF) is classified as acute (ALF), chronic (CLF) or acute-on-chronic (ACLF) [1–3]. In addition to the primary hepatic origin of LF, “secondary” LF may result from non-hepatic diseases such as sepsis [4]. All entities of LF are associated with prolonged hospital stay, increased costs and substantial mortality. LF of any origin frequently results in secondary organ dysfunction, most commonly leading to renal, circulatory and cerebral failure. Particularly in ACLF the number of organ failures is associated with poor short-term outcome approaching a 28-days- mortality of about 80% in ACLF if three or more organs fail [5]. In critically ill patients, “secondary” LF contributes more to mortality than any other organ failure, as demonstrated by a large prospective multi-centric cohort study [4]. Furthermore, any LF can progress to an irreversible state which requires liver transplantation (LTX). Because of the limited number of donor organs and contraindications to transplantation in a substantial number of patients, extracorporeal liver support (ELS) is considered an appealing concept to avoid transplant in case of reversible LF or as a “bridge to transplant”. This is further emphasized by a strong pathophysiological rationale to remove toxic mediators which frequently result in secondary failure of other organs. Several concepts including single pass albumin dialysis SPAD [6, 7], Molecular Adsorbent Recirculating System (MARS;[7–16]), plasma exchange [17, 18], fractionated plasma separation and adsorption [19] and bio-artificial devices [20, 21] have been investigated. For most of these procedures, improvement in secondary endpoints has been demonstrated in pre-clinical trials and clinical case series [6, 22, 23]. However, benefits regarding mortality or other strong endpoints have not been proven in a randomized controlled trial. With regard to multiple confounders and modifiers of outcome in this particular group of patients, a large number of patients would be required for an appropriately powered randomized controlled trial (RCT) using the currently available devices. These large numbers of patients for such a RCT are unlikely to be achieved due to treatment costs and organizational expenses. Therefore, optimization of ELS to increase its effect size and safety as well as offering additional features for advanced multi-organ support to enhance applicability might help to validate the clinical usefulness of advanced ELS.

**Fig. 1:**
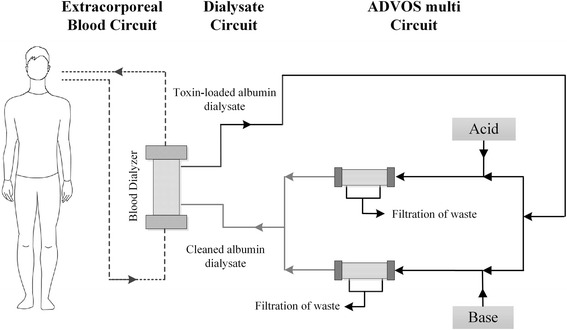
The advanced organ support (ADVOS) device

The ADVanced Organ Support (ADVOS) procedure is a newly introduced ELS (ADVOS multi, manufactured by Hepa Wash GmbH Munich, Germany), which potentially offers multi-organ support facilities. Rapid de-toxification and continuous regeneration of toxin-binding albumin is provided by tertiary circuits, which alter the binding capacities of albumin by biochemical (changing of pH) and physical (change of temperature) modulation of the effluent dialysate (Fig. 1; see Materials and Methods). Feasibility and effective detoxification have been shown in an animal study [24]. However, so far there were no reports on ADVOS treatment in humans.

Therefore, it was the aim of our study to investigate the efficacy of the elimination of bilirubin, creatinine and BUN in the first 14 patients ever treated with the ADVOS procedure.

## Methods

### Patients

The initial concept to evaluate the ADVOS procedure comprised two separate randomized trials including patients with ACLF (HEPATICUS-1/Trial-Registration: NCT01079091) and with “secondary” LF due to sepsis, multi-organ failure or acute LF without cirrhosis (HEPATICUS-2/Trial-Registration: NCT01079104). A “run-in” phase with up to five patients per study was part of the concept. These patients were not randomized, but were treated according to the protocols for the treatment groups of HEPATICUS-1 and −2. This approach was approved by the local Ethics Committee (“Ethikkommission der Fakultät für Medizin der Technischen Universität München”, reference number 2695/10; 2696/10). After the run-in phase, the studies HEPATICUS-1 and HEPATICUS-2 have not been continued due to lack of feasibility of the trial-concept. This decision was also based on the results of recently published randomized trials on MARS and fractionated plasma separation and adsorption. Consequently, the power calculation for HEPATICUS-1 and −2 was reanalyzed. Using a more “conservative” – i.e. pessimistic - estimation of the effect-size also including the experiences from HELIOS and RELIEF suggested that up to more than 700 patients might be needed for each trial.

The local Ethics Committee agreed on the evaluation of the seven patients treated in the run-in-phase of these studies and of another seven patients who were treated after the ADVOS procedure had received the CE-certificate in July 2013 (Ethikkommission der Fakultät für Medizin der Technischen Universität München”, reference number 18/15). The need for informed consent was waived for the patients after CE certification due to the retrospective design of the study regarding those seven patients. Informed consent was obtained from the seven patients included in the HEPATICUS-trials or their legal representatives.

The study was conducted in an eight-bed general intensive care unit of a German university hospital. Patients for the HEPATICUS-trials were included between October 2012 and August 2012.

### Inclusion criteria

Three patients of the run-in phase and six out of seven patients included after CE-certification had ACLF according to the CANONIC criteria [3, 25] and fulfilled all mandatory criteria of the HEPATICUS-1 trial:

1. Documented clinical or histological evidence of cirrhosis AND
2. Acute decompensation in previously stable cirrhotic liver disease AND
3. Bilirubin ≥2 mg/dl AND
4. SOFA score ≥9 [26] calculated after 12 h of optimal medical therapy AND
5. Patient is in the intensive care unit AND
6. Informed consent of the patient or the legal representative AND
7. Age of 18 years or older AND
8. Enrollment of patients within 96 h of fulfilling inclusion criteria 1–5

The inclusion criteria 6 and 8 were not mandatory for the seven post-CE certification patients.

Five patients suffered from “secondary” liver failure defined as acute liver failure of non-hepatic (e.g. sepsis, shock) or hepatic (e.g. ASH) origin in patients without evidence of chronic liver impairment. These patients fulfilled all mandatory criteria of the HEPATICUS-2-trial:

1. Patient is in the intensive care unit AND
2. Bilirubin ≥2 mg/dl AND
3. SOFA score ≥9 calculated 12 h after initial resuscitation measures AND
4. Signed informed consent of the patient or the legal representative AND
5. Age of 18 years or older AND
6. Enrollment of patients within 96 h of fulfilling inclusion criteria [1–5].

Criteria 4 and 6 were not mandatory for the post-CE certification patients

For exclusion criteria see Table 1

**Table 1:** Exclusion criteria of HEPATICUS-1 and of HEPATICUS-2 trial

Exclusion criteria for both HEPATICUS-1 and HEPATICUS-2	
Patients, whose mortality approaches 100%, or who are not likely to benefit from treatment (intervention with ADVOS is likely to be futile)	
Patients, whose current medical condition does not allow treatment with any extracorporeal procedure	
Potential conflict with good clinical practice (GCP) or with the declaration of Helsinki	
P_a_O_2_/F_i_O_2_ ≤ 100 mmHg (respiratory SOFA score of 4)	
Patient testament excludes the use of life-prolonging measures	
Post-operative patients whose liver failure is related to liver surgery	
Participation in another clinical study	
Patients diagnosed with Creutzfeldt-Jakob disease	
Pregnancy	
Weight ≥120 kg	
Uncontrolled seizures	
Mean arterial pressure ≤50 mmHg despite conventional medical treatment	
Active or uncontrolled bleeding	
Untreatable extrahepatic cholestasis	
Patients with MELD-score of 40	
Exclusion criteria specific for HEPATICUS-1	Exclusion criteria specific for HEPATICUS-2
Patients with creatinine >5 mg/dl or urine output <200 ml/day (renal SOFA-score of 4)	Patient with known history of chronic liver disease
Patients who receive a vasopressor support of Dopamine >15 μg/kg/min or epinephrine >0.1 μg/kg/min or norepinephrine >0.1 μg/kg/min (cardiovascular SOFA-score of 4)	

### The ADVOS procedure

The ADVOS multi device designed for extracorporeal liver and kidney support was used for all treatments. The device is composed of three circuits: A *blood circuit* to perfuse a commercially available dialyzer (FX 80; Fresenius Medical Care; D-61352 Bad Homburg; Germany) was established using a conventional double-lumen dialysis catheter (Gambro Gam Cath Dolphin; Gambro Hospal GmbH, Gröbenzell, Germany). Catheters with a length of 250 mm and a diameter of 13 F were used for femoral access and catheters with a length of 150–175 mm and a diameter of 13 F were used for internal jugular access, respectively. The primary and secondary circuits were established using a commercially available CRRT device (CF 200, Infomed SA, Geneva, Switzerland).

The *second circuit* was perfused by the albumin dialysate running on the dialysate side of the dialyzer *in parallel* with the blood flow. According to the information of the manufacturer several ex-vivo experiments had shown that for the elimination of *protein-*bound toxins concurrent flow is not inferior to countercurrent flow. The recycled albumin containing dialysate has a volume of approximately 2 liters. The composition of albumin dialysate was similar to a conventional hemodialysis dialysate with the only exception of addition of human albumin with an end-concentration of 2–4%.

The *third circuit* represents the proprietary ADVOS procedure and aims at regeneration and detoxification of toxin-loaded albumin. In this ADVOS circuit, the toxin-loaded albumin effluent from the dialyzer is branched into two parts. Each part undergoes a change of pH by adding acid or base before passing through the dialyzers resulting in a release of albumin-bound toxins in the dialysate. The unbound toxins are removed by a tertiary filtration process. Finally, the acidified and the alkalinized albumin dialysates converge so that an albumin dialysate with physiological pH (range 6.9–7.6) is generated again. In addition to biochemical modulation of the binding capacities of albumin, the ADVOS procedure also provides physical altering of albumin binding by heating and cooling of the dialysate. The primary temperature modulation can be performed by cooling of the dialysate to 28°. To keep this modulation “regional” the re-transfused blood is re-warmed to the pre-treatment body-temperature. Irrespective of our study – in which no systemic modulations of blood temperature were performed – this feature also allows for modulation of the body temperature (heating or cooling of the patient), if this is considered useful within the multi-organ support facilities of the ADVOS device.

Finally, the de-toxified dialysate containing unloaded albumin re-circles to the affluent connector of the dialysate compartment of the dialyzers.

### Anticoagulation

Anticoagulation was performed as required for conventional dialysis and modified for the patients’ individual coagulation state at the discretion of the treating physician.

### Pre- and post-dilution

No pre- or post-dilution was used during any treatment session.

### Length of treatment session

For ethical reasons and to optimize the individual therapeutic effect, there was no strictly pre-defined treatment period. Regarding the available experience with other devices for liver support the protocol aimed at treatment periods of 6–12 h which were achieved in the majority of first treatment sessions (see Table 3). Taking into consideration the disease severity of most of the patients, the treatment periods were modified according to other diagnostic or therapeutic requirements and according to the individual condition of the patient.

During ADVOS therapy no additional renal replacement therapy (serial or in parallel) was performed.

### Endpoints

With regard to efficacy and “proof of concept”, a comparison of pre- and post-treatment values of bilirubin, creatinine and blood urea nitrogen (BUN) was chosen as the primary endpoint. These blood analyses were performed immediately before connection and after disconnection to the ADVOS procedure. Due to the markedly different number of treatment sessions (1–101), analyses regarding the primary endpoint was restricted to the first treatment session in each patient.

### Statistics

The Wilcoxon-Test for paired samples was used to detect significant treatment effects. Spearman correlation was calculated to analyze the association of absolute and relative decreases in bilirubin, creatinine and BUN. P-values <0.05 were considered as statistically significant. All statistical tests were performed using IBM SPSS Statistics 23 (SPSS Inc., Chicago, IL, USA).

## Results

### Patients’ characteristics

The patients’ characteristics are shown in Table 2.

**Table 2:** Patients’ baseline characteristics

Patient	Study	Type of Liver failure (LF)	Aetiology	Precipitating event	Age [years]	Gender	CANONIC [ACLF-grade]	Child-Pugh [points]	MELD [points]	CLIF-SOFA [points]	Acute renal failure
P1	Post-CE	acute-on-chronic LF	alcoholic	SBP	59	female	3	12 (C)	40	12	+
P2	Post-CE	acute-on-chronic LF	alcoholic	ASH	52	female	2	12 (C)	26	14	+
P3	Post-CE	acute-on-chronic LF	alcoholic	ASH	53	male	3	13 (C)	37	15	+
P4	Post-CE	acute-on-chronic LF	alcoholic	Infection	61	male	3	11 (C)	35	15	+
P5	Post-CE	acute-on-chronic LF	alcoholic	Ischemia	68	female	3	12 (C)	24	18	+
P6	Post-CE	acute-on-chronic LF	alcoholic	ASH, sepsis	48	female	3	12 (C)	44	13	+
P7	HEPATICUS-1	acute-on-chronic LF	alcoholic	GI-bleeding	54	male	3	14 (C)	29	14	+
P8	HEPATICUS-1	acute-on-chronic LF	alcoholic	GI-bleeding	38	male	3	13 (C)	32	17	+
P9	HEPATICUS-1	acute-on-chronic LF	alcoholic	ASH	28	male	3	11 (C)	34	13	+
P10	HEPATICUS-2	“Secondary” LF	Sepsis	n.a.	66	female	n.a.	n.a.	19	18	+
P11	HEPATICUS-2	“Secondary” LF	ASH	n.a.	39	male	n.a.	n.a.	39	9	+
P12	HEPATICUS-2	“Secondary” LF	Drug-induced	n.a.	73	male	n.a.	n.a.	38	15	+
P13	HEPATICUS-2	“Secondary” LF	ASH	n.a.	56	female	n.a.	n.a.	35	14	+
P14	Post-CE	“Secondary” LF	Sepsis	n.a.	67	male	n.a.	n.a.	40	18	+
Mean ± SD or number [%]					54 ± 13	8 male; 6 female	2.9 ± 0.3	12 ± 1	34 ± 7	15 ± 3	100%

*ASH* alcoholic steatohepatitis, *SBP* spontaneous bacterial peritonitis, *MELD* model of end-stage liver disease

*CLIF-SOFA* chronic liver-failure sequential organ failure assessment, *CANONIC* CLIF acute-on-chronic liver failure in cirrhosis

*n.a* not applicable

All patients were critically ill with a high predicted mortality according to different scores of HF and general prognostic scores for critically ill patients: All but one patient with ACLF had the highest score of the CANONIC-ACLF staging (stage C) [3]. The MELD-score [27] in these patients was 34 ± 7 points. The mean CLIF-SOFA [3] was 15 ± 3 points. Only two patients were listed for liver transplant. None of the patients underwent liver transplantation due to unavailable organ or acute contraindications for transplantations.

The most frequent precipitating event of ACLF was acute alcoholic hepatitis in four of nine cases. Two more cases of ACLF were related to infectious complications. Another two patients without ACLF suffered from first episodes of alcoholic steatohepatitis without evidence of cirrhosis which precludes listing for liver transplantation for six months according to German allocation laws. The other three cases of secondary LF were induced by sepsis. Among those patients were two patients with malignancies.

All 14 patients suffered from acute renal failure according to the AKIN criteria. Seven of 14 patients had undergone renal replacement therapy before the first ADVOS treatment.

### Primary endpoint: treatment efficacy

**Fig. 2:**
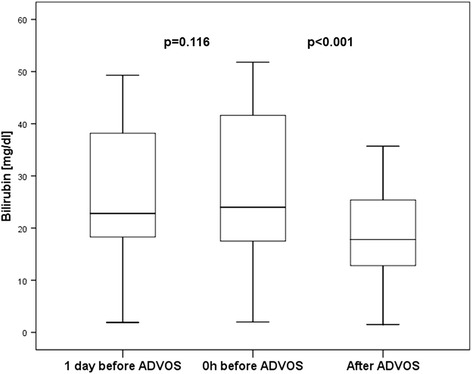
Boxplots depicting the time course of serum bilirubin

**Fig. 3:**
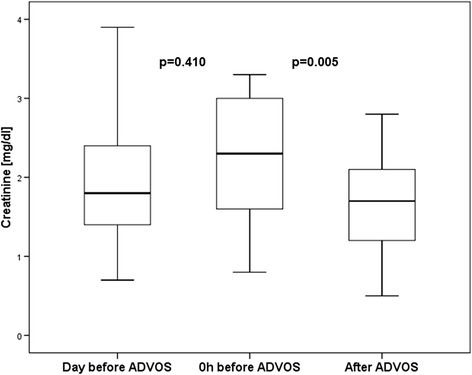
Boxplots depicting the time course of serum creatinine

**Fig. 4:**
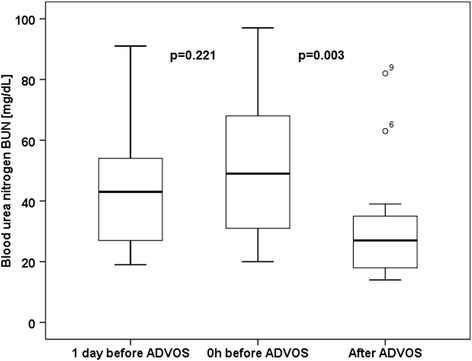
Boxplots depicting the time course of serum BUN

The 14 first treatment sessions were performed for a mean of 575 ± 193 min without interruption or need for disconnection due to technical reasons or adverse events related to the procedure. The serum levels of both protein-bound and water-soluble toxins were significantly decreased during treatment with the ADVOS procedure: Se5rum bilirubin levels were significantly decreased by 32% after a single ADVOS procedure (17.7 ± 10.5 mg/dl vs. 26.0 mg/dl ± 15.4 mg/dl; *p* = 0.001). Similarly, serum creatinine (1.6 ± 0.7 mg/dl vs. 2.2 ± 0.8 mg/dl; *p* = 0.005) and BUN (31.1 ± 20.29 mg/dl vs. 49.4 ± 23.3 mg/dl; *p* = 0.003) were significantly lowered by a mean of 27% and 37% by a single ADVOS treatment, respectively (Table 3; Figs. 2, 3 and 4).

**Table 3:** Treatment data and outcome

	Treat-ment time [min] [19]	Serum bilirubin [mg/dl]				Serum creatinine [mg/dl]				Serum BUN [mg/dl]				No. of treatments	28d survival
		Day before ADVOS	Before start of ADVOS	After ADVOS	Delta [mg/dl]	Day before ADVOS	Before start of ADVOS	After ADVOS	Delta [mg/dl]	Day before ADVOS	Before start of ADVOS	After ADVOS	Delta [mg/dl]		
Patient															
P1	745	37.4	41.1	25.4	−15.7	0.7	0.8	0.5	−0.3	59	76	28	−48	19	-
P2	800	22.8	24.0	12.8	−11.2	1.0	1.0	0.9	−0.1	26	25	14	−11	10	-
P3	730	20.8	22.3	14.1	−8.2	1.8	1.6	1.5	−0.1	21	20	18	−2	7	-
P4	770	16.3	15.9	13.2	−2.7	2.4	1.9	1.5	−0.4	27	20	18	−2	29	-
P5	800	n.d.	11.6	9.2	−2.4	n.d.	2.8	1.2	−1.6	n.d.	45	13	−32	3	-
P6	600	43.5	41.6	34.2	−7.4	3.9	3.3	2.2	−1.1	91	97	63	−34	22	+
P7	720	3.1	5.3	3.9	−1.4	1.7	3.0	2.1	−0.9	19	33	22	−11	17	+
P8	420	21.6	20.7	17.8	−2.9	1.3	2.0	2.8	+0.8	44	69	82	+13	9	-
P9	570	26.4	26.2	20.3	−5.9	1.9	2.3	1.7	−0.6	43	51	35	−16	6	+
P10	300	1.9	2.0	1.5	−0.5	1.7	1.3	0.7	−0.6	54	31	16	−15	1	-
P11	240	40.4	41.6	29.0	−12.6	3.0	3.2	2.0	−01.2	35	40	27	−13	101	+
P12	510	18.3	17.5	10.9	−6.6	1.4	2.9	2.1	−0.8	40	49	27	−22	3	-
P13	360	38.2	42.1	19.9	−22.2	3.0	2.3	1.2	−1.1	79	68	33	−35	11	+
P14	480	49.3	51.8	35.7	−16.1	3.0	3.0	2.2	−0.8	54	67	39	−28	1	-
Mean ± SD Number [%]	575± 193	26.2± 14.9	26.0± 15.4	17.7± 10.5	−8.3± 6.5	2.0± 0.9	2.2± 0.8	1.6± 0.7	−0.6± 0.6	46± 22	49± 23	31± 20	−18± 16	17± 26 [1–101]	5/14 [36%]

*ADVOS* advanced organ support

*BUN* blood urea nitrogen

*n.d*. not done

*Delta* absolute changes when comparing values immediately before and after the first ADVOS session

### Treatment efficacy at different starting values

There was a significant correlation between the baseline serum levels of bilirubin, creatinine and BUN with their absolute reduction induced by the first ADVOS treatment (*r* = 0.911, *p* < 0.001; *r* = 0.756, *p* = 0.002; *r* = 0.644, *p* = 0.013), respectively (see also Table 3). By contrast, the relative changes in serum bilirubin, creatinine and BUN were not associated to their baseline levels (*r* = 0.383 *p* = 0.177; *r* = 0.236 *p* = 0.417; *r* = 0.247 *p* = 0.395).

### Safety and outcome

In summary, the ADVOS treatment was hemodynamically well tolerated during a mean of 9.7 ± 3.5 h of therapy. Overall, 239 treatments were carried out (17 ± 26; 1–101 treatments per patient). None of the treatment sessions had to be interrupted due to complications that were likely related to the ADVOS procedure. The 28-days survival rate was 5/15 (36%). Finally, two patients (14%) were discharged home.

## Discussion

At present, there are three procedures of extracorporeal liver support based on albumin-dialysis used in clinical practice. Albumin-dialysis aims at the elimination of albumin-bound endogenous toxins such as bile-acids that accumulate in case of LF via a semipermeable membrane. For this purpose, albumin has to be added to the dialysate in a final concentration between 2% and 20% [6, 7, 10, 11, 28]. In SPAD, dialysate flow rates range between 0.5 L/h and 2 L/h. SPAD is easily available, can be performed with standard renal replacement therapy (RRT) devices and has the advantage of “naïve” albumin in the dialysate which provides maximum detoxification capacities of the dialysate [6]. However, with repeated treatment sessions up to 24 h and a market price of commercially available 20% human albumin solutions up to 150€ per 100 mL, a total albumin cost of approximately 1500€ per session has been estimated [6]. Therefore, attempts have been made to recirculate albumin after regeneration (cleansing). MARS, the second type of albumin-dialysis, helps to save albumin resources. Its efficacy in elimination of albumin bound toxins has been demonstrated in several case-series and studies [7, 10, 11, 28]. While MARS uses a charcoal column and an anion exchange resin column to unload albumin-bound toxins and to regenerate albumin, the recently introduced ADVOS procedure continuously unloads and regenerates albumin by intermittent alteration of its binding capacities [7, 10, 11, 24, 28]. This is achieved by biochemical (changing of pH) and physical (change of temperature) modulation of the effluent dialysate. In addition to its features as ELS, ADVOS – at least from a theoretical viewpoint - offers the advantage of multi-organ support by providing the potential to modulate the acid–base-balance and the body temperature of the patient. While MARS is usually performed with 600 mL of 20% albumin, this amount is further reduced in the ADVOS procedure to 200 mL [24].

Nevertheless, similar to MARS, regeneration of albumin carries the potential for its denaturation and loss of efficacy.

Our study in 14 patients primarily analyzed the efficacy of elimination of protein-bound and water-soluble toxins by the ADVOS procedure. As demonstrated in Table 2 and Table 4, ADVOS significantly reduced serum bilirubin, creatinine and BUN by 32%, 27% and 37%, respectively. Although cross-comparison with data from other recent studies with different inclusion criteria and populations has to be performed with caution, our data suggest at least comparable elimination capacities of ADVOS to those reported for MARS, fractionized plasma separation and adsorption (Prometheus) and SPAD in the most recent studies (Table 4). Furthermore, it has to be kept in mind that these elimination rates were achieved by a single ADVOS treatment compared to repeated (two up to eight) sessions performed in the reference studies with the other devices. Treatment periods between seven and ten hours were comparable for all methods.

**Table 4:** Comparison of patients’ characteristics and elimination efficacy

Trial/Reference	Banares [10]	Kribben [19]	Sponholz MARS [7]	Sponholz SPAD [7]	Own study
Device	MARS	Prometheus	MARS	SPAD	ADVOS
Patients’ characteristics					
Clinical setting	19 ICUs	10 ICUs	Surgical ICU		Medical ICU
Type of LF					
- ACLF	100%	100%	56%		64%
- ALF	-	-	28%		-
- “secondary” LF	-	-	-		36%
- Graft failure	-	-	16%		-
(CLIF)-SOFA	8 ± 3	10 ± 3	13 ± 4		15 ± 3
MELD	26 ± 8	28 ± 10			34 ± 7
Child-Pugh	11 ± 2	12 ± 1			12 ± 1
Efficacy analysis					
No. of treatments for efficacy analysis	4	8 (mean)	2.2 (mean)	2.2 (mean)	1
Delta-Bilirubin relative	−26% (day 4)	−23% (day 28)	−23%	−23%	−32%
Delta-Bilirubin absolute [mg/dL]	−8.7 (day 4)	−6 (day 28)	−4.3	−4.2	−8.3
Delta-creatinine relative	−20% (day 4)	−13% (day 28)	−18%	+5%	−27%
Delta-creatinine absolute [mg/dL]	−0.3 (day 4)	−0.2 (day 28)	−0.4	+0.1	−0.6
Delta-BUN relative	n.d.	n.d.	−9%	+5%	−37%
Delta-BUN absolute [mg/dL]	n.d	n.d.	−12	+8	−18
Maximum number of treatments	10	11	4	4	101

Similarly, the interpretation of feasibility and safety data is limited due to the small number of patients treated and due to the lack of a direct control group. However, our conclusions are supported by the high number of total treatment sessions that was done reaching up to 101 sessions in one single patient.

Although we have to clearly admit that this study was not powered for mortality analysis and lacked a control group, the final outcome of the patients is worth mentioning. Due to the lack of direct controls, the outcome of our patients has to be compared to the data available from large scoring databases (e.g. CLIF-SOFA). These databases provide validated outcome estimates for patients comparable those in our study. At first glance, survival was poor with 28-days and in-hospital mortality rates of 65% and 86%, respectively. This might be related to several reasons: As supported by all scoring systems investigated, the patients were more severely ill compared to the randomized trials summarized in Table 4. All but one patient with ACLF had Grade 3 according to the CANONIC criteria, which has been associated in a recent study with 28-days- and 3-months-mortality rates of 83.6% and 87.3%, respectively [5]. For the mean CLIF-SOFA score of 15 points in our patients, the same study predicts a high 28-days-mortality approaching 90%. Furthermore, two of our patients suffered from malignancies, and in at least five patients active alcoholism was suggested by the presence of ASH. Ongoing alcoholism - precluding LTX according to German allocation rules – was also evident in three patients with ACLF. Two patients suffered from “secondary” (see above) liver failure due to severe sepsis and was classified as non-transplantable. This may explain why only two patients were listed for liver transplantation. None of these patients underwent LTX during the observation period. These individual medical histories emphasize that extracorporeal liver support was initiated in most of our patients as a rescue treatment, since LTX was not feasible.

### Strengths and limitations of the study

This study demonstrated feasibility and efficacy of a new approach to ELS. To the best of our knowledge a total number of 101 treatment sessions in one single patient has not been reported for any other procedure of ELS. Elimination rates for surrogate markers were among the highest ever reported (Table 4) suggesting a high efficacy during the first treatment. The patients included are heterogenous regarding the aetiology of liver failure and also regarding the number of treatment sessions. This heterogeneity could be considered as a methodological limitation. On the other hand, this heterogeneity might give first hints on potential indications and general applicability of the procedure in different groups of patients with severe liver failure. Furthermore, any reporting bias could be avoided, since we reported on all patients who have ever been treated with ADVOS in our ICU. Despite feasibility, safety and efficacy of the intervention, the overall outcome was poor, which might be related to the use of ADVOS as a rescue therapy in this study which predominantly included patients without the option to undergo LTX. Another limitation is the low number of patients included that precludes more robust conclusions regarding the outcome. Conclusions regarding the outcome are further limited by the lack of a randomized control group or matched controls. Finally, even in case of randomized controls the statistical power would be low due to the restricted number of comparisons.

## Conclusion

ADVOS efficiently eliminates water- and protein-bound toxins in humans with LF. ADVOS is feasible in patients with advanced LF which is emphasized by a total number of more than 100 treatment sessions in one single patient.

## Abbreviations

ACLF: Acute-on-chronic liver failure
ADVOS: Advanced organ support
BUN: Blood urea nitrogen
CANONIC: CLIF acute-on-chronic liver failure in cirrhosis
CLIF: Chronic liver failure
ELS: Extracorporeal liver support
FPSA: Fractionated plasma separation and adsorption
LF: Liver failure
LTX: Liver transplantation
MARS: Molecular adsorbents recirculating system
MELD: Model for end-stage liver disease
RRT: Renal replacement therapy
SPAD: Single pass albumin dialysis
